# Health Impacts of Urban Bicycling in Mexico

**DOI:** 10.3390/ijerph18052300

**Published:** 2021-02-26

**Authors:** David Rojas-Rueda

**Affiliations:** Department of Environmental and Radiological Health Sciences, Colorado State University, Fort Collins, CO 80523, USA; David.Rojas@colostate.edu; Tel.: +1-(970)-491-7038; Fax: +1-(970)-491-2940

**Keywords:** bicycling, transport, Mexico, health impact assessment, environmental health

## Abstract

**Background:** Bicycling has been associated with health benefits. Local and national authorities have been promoting bicycling as a tool to improve public health and the environment. Mexico is one of the largest Latin American countries, with high levels of sedentarism and non-communicable diseases. No previous studies have estimated the health impacts of Mexico’s national bicycling scenarios. **Aim:** Quantify the health impacts of Mexico urban bicycling scenarios. **Methodology:** Quantitative Health Impact Assessment, estimating health risks and benefits of bicycling scenarios in 51,718,756 adult urban inhabitants in Mexico (between 20 and 64 years old). Five bike scenarios were created based on current bike trends in Mexico. The number of premature deaths (increased or reduced) was estimated in relation to physical activity, road traffic fatalities, and air pollution. Input data were collected from national publicly available data sources from transport, environment, health and population reports, and surveys, in addition to scientific literature. **Results:** We estimated that nine premature deaths are prevented each year among urban populations in Mexico on the current car-bike substitution and trip levels (1% of bike trips), with an annual health economic benefit of US $1,897,920. If Mexico achieves similar trip levels to those reported in The Netherlands (27% of bike trips), 217 premature deaths could be saved annually, with an economic impact of US $45,760,960. In all bicycling scenarios assessed in Mexico, physical activity’s health benefits outweighed the health risks related to traffic fatalities and air pollution exposure. **Conclusion:** The study found that bicycling promotion in Mexico would provide important health benefits. The benefits of physical activity outweigh the risk from traffic fatalities and air pollution exposure in bicyclists. At the national level, Mexico could consider using sustainable transport policies as a tool to promote public health. Specifically, the support of active transportation through bicycling and urban design improvements could encourage physical activity and its health co-benefits.

## 1. Introduction

The United Nations has reported that more than 50% of the global population lived in urban settings in 2018, and the urbanization trend is expected to increase in the coming years [1]. Urban and transport planning has been suggested as a critical health determinant, impacting physical activity, air and noise quality, traffic safety, blue and green spaces, among others [2,3]. Specifically, bicycling has been suggested as a tool to promote physical activity [4,5,6]. 

Sedentarism is one of the leading risk factors for mortality worldwide [7]. The global prevalence of insufficient physical activity in 2016 was 23%, and the Latin American region had the highest prevalence of insufficient physical activity (39%) [8]. Mexico is the second most populated country in the Latin American region, with 127 million inhabitants [9], with more than 80% of its population living in urban areas [1]. Mexico has reported 29% of the population has insufficient physical activity [8].

Active transport policies have been promoted extensively in Latin America, being the open street programs (where main streets in cities are closed for walking and cycling), one of the most known active transport policy originated in Latin America [10,11,12]. Although bicycling has played an essential role in personal mobility around the world, current trends show that motorized traffic is gaining more relevance [13]. Compared to other modes of transportation, bicycles offer a convenient and affordable transport option that could capture a higher proportion of urban transport passengers than is currently the case [13].

Previous studies have estimated the health impacts of local bicycling transport scenarios, but most of them have been focused on developed countries [14,15,16]. To our knowledge, no study has assessed the health impact of bicycling scenarios in Mexico. This study aims to estimate the health impacts, risks, and benefits of Mexico bicycling scenarios at the national level.

## 2. Methodology

### 2.1. Study Design and Data Collection

This study follows a quantitative health impact assessment (HIA) approach, assessing bicycling scenarios in the urban population in Mexico. Transport data were collected from the “Global High Shift Cycling” study [13]. The “Global High Shift Cycling” study provides bicycling data at a national level, describing transport patterns such as trips per person per day, trip length, kilometers traveled by a person, and mode of transport (Table 1). Methods and descriptions of the “Global High Shift Cycling” study have been reported elsewhere [13]. National population data were obtained from the United Nations population forecast [1]. Mortality rates by age and country were collected from the year 2017, which was reported by the Global Burden of Disease (GBD) project [17]. Air pollution data of particulate matter less than 2.5 micrometers of diameter (PM2.5) annual average national concentration was collected from the World Health Organization (WHO) Global Ambient Air Quality Database [18]. National annual traffic fatalities by mode of transport were collected from the Road Safety Annual Reports [19] and the global observatory data from the World Health Organization from years 2009 to 2018 [20]. National physical activity data in metabolic equivalent of task (MET) were collected from scientific publications [21,22]. Dose–response functions used in this quantitative Health Impact Assessment (HIA) for physical activity and air pollution on all-cause mortality were collected from the published meta-analysis [23,24].

### 2.2. Scenarios

**Figure 1:**
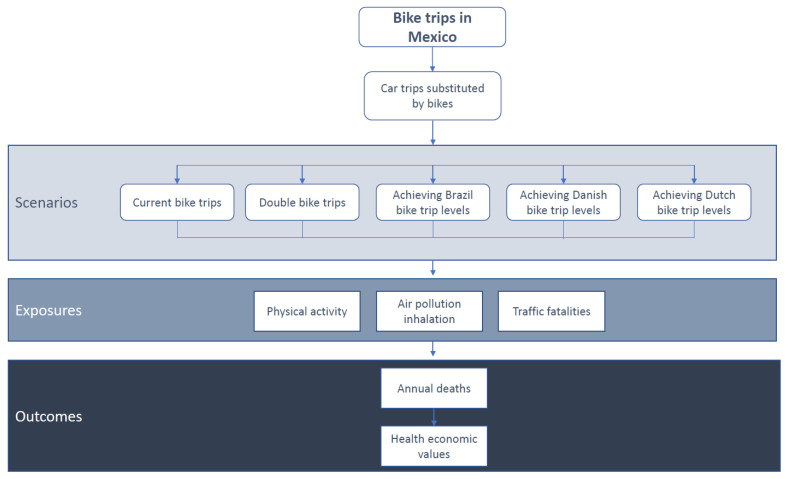
Conceptual framework of the study.

Five scenarios were included in this study (Figure 1): (a) current bike levels in Mexico (based on the bike trips reported at the national level for adults in urban population, 1.07%) [13]; (b) double the national bike-share (assuming as transport goal doubling the current levels of bike trips, 2.13%); (c) arriving at bike levels reported in Brazil (Brazil was the Latin America country with the largest bike mode share reported, 3%) [13]; (d) achieving the Danish bike levels (Denmark is reference country for bicycling, 16%) [25]; and (e) achieving the Dutch bike levels (The Netherlands is the country with the largest bike share in the world, 27%) [26]. All the scenarios assumed an 8% car-bike substitution based on the average reported substitution among 26 cities worldwide [14,27,28,29,30]. All scenarios assumed a conservative average bike trip distance in Mexico of 2 km.

### 2.3. Quantitative Model

A quantitative health impact assessment approach was followed to estimate the number of annual premature deaths related to each scenario and health determinant (Figure 1). All-cause mortality was estimated considering three different health determinants (physical activity, road traffic fatalities, and air pollution (PM2.5)). The “TAPAS (transportation, air pollution, and physical activities) tool” developed and used in previous quantitative HIA was used to estimate the health impacts in this study [6,14]. A detailed description of the TAPAS tool methods has been reported in the supplemental material and elsewhere [6,14,31,32]. TAPAS tool is a quantitative HIA run on Microsoft Excel for Office 365, version 2008 (Microsoft, Redmond, USA, 2020). The dose–response functions used in the TAPAS tool, between physical activity, PM2.5, and all-cause mortality, were selected from meta-analyses of cohort studies from adult populations. The risk estimated from traffic fatalities by kilometer traveled was obtained from national transport and health data. Levels of each determinant were estimated for each country and scenario. An all-cause mortality relative risk (RR) was estimated for each health determinant and scenario and transformed into a population attributable fraction (PAF). Using the Mexico mortality rate for adults (20–64 years old) and the national urban adult population (20–64 years old) in each scenario, the number of expected premature deaths was estimated for each scenario. Finally, the PAF from each scenario was multiplied with the corresponding expected number of premature deaths in the population to obtain the number of attributable premature deaths. For the economic assessment, the value of statistical life was used to estimate the economic impacts of preventing deaths in each scenario, using the value of statistical life reported for Mexico (US $210,880) [33].

#### 2.3.1. Physical Activity

The physical activity level was estimated based on the trip duration, trip frequency, and physical activity intensity, using the metabolic equivalent of task (MET) (Table 1). The physical activity was defined as 6.8 METs for bikes and 2 METs for car travelers. The relative risk of all-cause mortality was based on the dose–response function (DRF) provided by a meta-analysis of cohort studies (RR = 0.81 (0.76–0.84) for each increment of 8.6 METs, with a power transformation of 0.25)) [24], assuming a non-linear DRF. The physical activity assessment considers the basal levels of physical activity in the Mexican population [21,22] to estimate the relative risk for each scenario before being translated into a population attributable fraction and then to the estimated attributable premature deaths (see Supplemental Material Figures S1 and S2).

#### 2.3.2. Air Pollution

The air pollution assessment focused only on the exposure to particulate matter with a diameter < 2.5 μm (PM2.5), which has shown a strong association with all-cause mortality [34,35,36]. We obtained the annual average PM2.5 concentrations in Mexico, using the World Health Organization database of air quality [18] (Table 1). We estimated the concentration of PM2.5 in each microenvironment (bike and car), using background/car or bike ratios provided by a previous meta-analysis [37], following a similar approach as reported in previous studies [14,16,31] (see Supplemental Material Figure S3 and Tables S1–S3). The inhaled dose was estimated using the minute ventilation according to the intensity of physical activity (in METs) in each mode of transport (bike and car), PM2.5 concentration in the mode of transport, and trip duration [14,16,31] (see Supplemental Material Tables S2 and S3). The DRF for PM2.5 and all-cause mortality from a meta-analysis were used (RR = 1.06 (1.04, 1.08)) for each increment of 10 μg/m3 of PM2.5) [23]. Finally, using the comparative risk assessment approach, we estimated the relative risk, population attributable fraction, and the expected number of premature deaths for each scenario, as reported before (see Supplemental Material Figure S3).

#### 2.3.3. Road Traffic Fatalities

The road traffic fatalities in Mexico were obtained from the annual traffic fatalities reported at the national level through transport mode from years 2009 to 2018 (Table 1). For each scenario, we estimated the number of kilometers traveled by car and bike. The expected traffic fatalities by mode of transport were estimated using the traffic fatalities per billion kilometers traveled and the distance traveled in each mode of transport [31,34]. Then a relative risk of traffic fatalities for cyclists compared with car drivers was estimated. The relative risk was translated to an attributable fraction and a final number of prevented premature deaths in each scenario (see Supplemental Material Table S1 and Figure S4).

## 3. Results

The national bike share in Mexico was 1.07% of all trips. We estimated an average of 2,068,750 daily bike trips among adults in urban settings in Mexico (Table 1). The number of bike trips per day (<2 km) was estimated to substitute car trips in Mexico where 165,500. In all the scenarios, the health benefits (in preventable deaths) of physical activity related to bicycling outweighed the health risks associated with traffic fatalities and air pollution inhalation (Table 2).

### 3.1. Impacts of Current Bicycling Levels in Mexico

**Figure 2:**
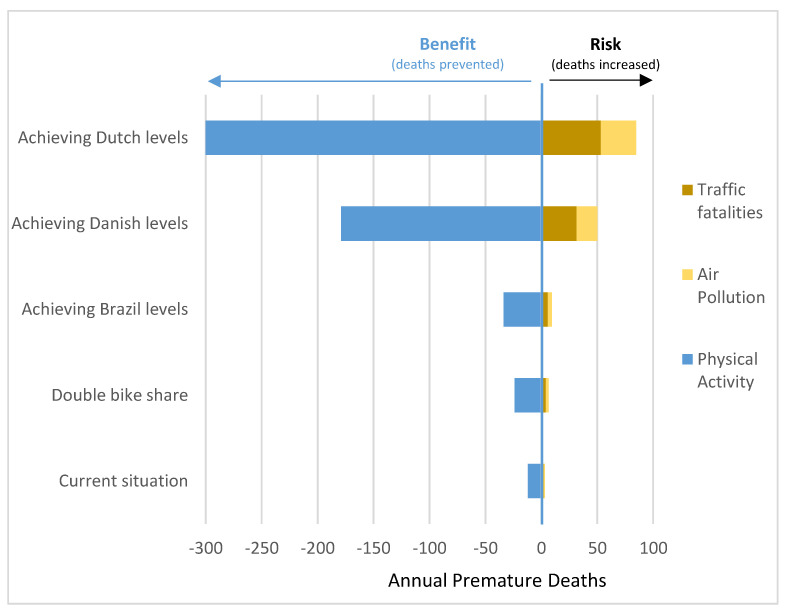
Risks and benefits of bicycling scenarios in Mexico, in annual premature deaths by scenario and risk factor.

It was estimated that the current levels of bike trips in Mexico (that are expected to substitute car trips, 165,500 trips per day) resulted in 9 (95% UI: 6–25) premature deaths avoided each year among the urban adult population. In terms of economic values, it was estimated that the current bike trips could result in US $1,897,920 annual health economic benefits related to mortality (Table 2). In terms of risks and benefits, traffic fatalities were estimated to increase 2 annual deaths and air pollution exposure 1 annual death. Physical activity resulted in the prevention of 12 annual deaths (Figure 2 and Supplemental Material Table S4)

### 3.2. Impacts of Future Bicycling Scenarios in Mexico

If Mexico doubles the current levels of bike trips to 2.13% (assuming similar trips substitution from cart to bike, that current levels), the annual premature deaths prevented could arrive at 17 (95% UI: 11–49), with an economical translation of US $3,584,960. If Mexico achieves the bike trip levels reported in Brazil (3%), the annual benefits could arrive at 24 (95% UI: 16–69), with an economic impact of US $5,061,120. If Mexico arrives at bike trip levels reported in Denmark (16% of bike trips), the health impacts will be translated into 129 (95% UI: 84–370) annual prevented deaths and US $27,203,520. Finally, suppose Mexico achieves bike trip levels similar to those reported by The Netherlands. In that case, the health impacts will be an annual reduction of 217 (95% UI: 142–625) prevented deaths, with an annual health economic benefit of US $45,760,960.

## 4. Discussion

This study found that bikes in Mexico have the potential to prevent up to 217 annual premature deaths if bike trip levels, similar to those reported in the Netherlands, are achieved with an annual economic benefit of more than 45 million US dollars (Table 1). In the current situation, bike trip levels in Mexico are expected to prevent 9 premature deaths each year (Table 1). In the five scenarios assessed, the health benefits (due to physical activity) outweighed the health risks (air pollution inhalation and traffic incidents) (Figure 2).

This is the first study assessing the health impacts of national bicycling scenarios in Mexico. This study included the 51,718,756 adult urban inhabitants in Mexico. This study includes five different bicycling scenarios comparing the current bike levels in Mexico with reference counties in Latin America (Brazil) and worldwide (Denmark and the Netherlands, the global reference countries for bicycling trends). This study provides a conservative estimation of the bicycle health impacts in Mexico. The analysis only includes a small portion of bike trips (those assumed to came from cars (8% of all bike trips)), assuming short trip distances 2 km, including only adult population (20–64 years old) and urban settings. The overall health impacts of bicycling in Mexico are expected to be larger if all bicycle trips and populations are counted. 

These results are in accordance with previous quantitative health impact assessment studies on bicycling scenarios using similar exposures (physical activity, air pollution, and traffic fatalities) [14,31,38,39,40]. A previous study in seven European cities found that achieving 10% of bike trips will prevent between 0 to 31 deaths in Antwerp and Vienna [38]. In this study was assumed that 28% of bicycling increments came from cars [38]. Another study on the health impacts of bike-sharing systems in Barcelona, Spain, was found that if 90% of the bike-sharing trips came from cars (around 38,000 trips per day), 12 deaths could be avoided each year [31]. Another study with more ambitious scenarios from six European cities estimated the health impacts of bicycling scenarios in Paris, Prague, Warsaw, Basel, and Barcelona [2]. In this study, the aim was to assess “what if” those cities achieve the bike share from Copenhagen (35%) [2]. In this case, the study estimated among 5 to 113 premature deaths prevented each year between the six European cities [2]. Unlike previous studies that have been focused on single cities [2,6,14,31], our study focused on the national urban populations, providing a broader perspective of policy scenarios in Lat America. Like previous studies, this analysis focused on car trip substitution, considering that sifting car trips to active transportation will have larger health benefits and important climate co-benefits [2].

This study found that the current bicycling levels in Mexico will benefit public health at a national scale, preventing nine premature deaths annually among adult urban populations (that are expected to shift from car to bike). Those results were also translated into economic impacts related to mortality, using the value of statistical life, a standard metric used by transport planners and engineers to measure traffic safety impacts. We estimated that Mexico’s current car-bike substitution levels have an economic benefit of up to 1.8 million US dollars annually. This study also included different hypothetical policy scenarios related to bike share. We selected four extra bike scenarios to compare “what if” Mexico increases their bike levels to double the current bike share (2.13%); or to those reported by Brazil (3%) that was the Latin American country with the highest bicycling levels; or to those reported in Denmark (16%) or the Netherlands (27%), the reference countries for bicycling around the globe. In those scenarios, the health benefits ranged between 17 to 217 annual premature deaths that could be prevented each year, with an economic benefit between 3.5 to 45 million US dollars annually (Table 1). These results highlight the importance of active transportation policies in Mexico and the potential of transport policies to support public health. 

Among the exposures included in this quantitative health impact assessment, physical activity produced the most considerable health impacts (Figure 2). Physical activity is well known as a health-protective factor for multiple diseases and causes of death, such as cardiovascular, metabolic, and mental diseases, among others [16]. Our analyses focused on all-cause mortality as a health outcome because it has been proposed as the best indicator of health impacts on active transport assessments compared to morbidity [6,16]. This analysis utilized the “TAPAS tool,” a quantitative health impact assessment tool for bicycling, walking, and public transport, reported in previous transport health impact assessments [2,6,14,31]. The “TAPAS tool” for bicycling estimated the health impacts of physical activity using a non-linear dose–response function (DRF) from a meta-analysis of cohort studies [24], and it was calibrated with the corresponded physical activity levels reported by the adult population in Mexico and applied to the exposure levels by each scenario. The non-linear function considers that those who already were physically active would gain fewer health benefits than those who are more sedentary. This non-linear approach results in a conservative result estimating fewer health benefits than using a linear DRF [2].

In this study, air pollution analysis only considers the exposure to PM2.5 inhalation during the trip. Although air quality improvements can be expected from changes in modal share, these health-related impacts were not in the scope of this study, and the study only focused on the PM2.5 exposure of bicyclists during the trip. PM2.5 was selected because it was expected to produce the largest health burden compared to other air pollutants such as NO2 or black carbon [6]. 

Traffic safety analysis was based on traffic fatalities. This study quantified fatal traffic incidents per billion kilometers traveled, using the reported national road safety estimates provided by the World Health Organization (WHO) [20]. This study only considered the traffic fatality risk by mode of transport (bike vs. car). It did not assess the impacts of other traffic risk factors such as the type of route or traveler demographics due to the lack of data available in these aspects. 

Our study was limited by data availability and the necessity to make assumptions to model likely scenarios. In terms of the scenarios modeled, we select national bicycling goals similar to those that already exist in other nations in Latin America and globally. However, one limitation is the transferability of the policy scenarios to the Mexican context. In Denmark and the Netherlands, geographical and social characteristics differ from the Mexican context (i.e., population density, transport infrastructure, or land use and cartography). Another limitation was the lack of specific modal shift (car to bike) data from Mexico. Thus, the data available from 26 cities from China, Europe, and the US was summarized to estimate the average percentage of bike trips that can shift from car trips [14,27,29,30]. Our estimates’ uncertainty was also assessed, providing uncertainty intervals composed of the input data’s variability (maximum and minimum) and the confidence intervals from the DRF from air pollution and physical activity. Another limitation in this study was the need to assume an average trip distance in Mexico. In this study, we selected 2 km as a conservative scenario. But a sensitivity analysis was conducted to estimate the health impacts in the five scenarios if a similar bike trip length (5 km) was used as reported in a previous study in Europe [38]. In this sensitivity analysis, we found that the health benefits of urban bike trips in Mexico could be estimated between 15 to 384 preventable annual deaths among the five scenarios (see Supplemental Material Tables S5 and S6). 

Furthermore, if national and local authorities improve traffic safety and air quality, in addition, to increase bike levels, more health benefits could be expected in Mexico. In all our scenarios, we assume only an 8% of car–bike trip substitution. This is of particular relevance because if authorities achieve attracting more car drivers and passengers to bicycles, the health benefits could increase largely in addition to the overall levels of bike trips. This study only considers a population between 20 and 64 years old. If policymakers and transport planners achieve the goal of attracting younger and older age groups to bicycles, the health benefits from bicycling in Mexico could be more extensive. As in many other countries, the aging process is also affecting the Mexican population [1]. Healthy aging starts with integrating a healthy lifestyle since the early stages of life, and bicycling could be used as a tool to promote healthy aging. Some general recommendations for policymakers and stakeholders to promote bicycling in Mexico are (a) the support of active transport policies, specifically on interventions to promote bicycling and reduce car driving; (b) support traffic safety and air quality improvements in urban settings in Mexico; and (c) improve data collection and quality improvement in terms of physical activity, traffic safety, air quality, and transport characteristics. For health practitioners, this study can help to dimension the relevance of transport policies to improve public health. Researchers should support local and national data collection on transport and health with a vision of harmonization and comparability. A summary of the policies needed to increase bicycling in Mexico is listed in Table 3.

## 5. Conclusions

The study found that bicycling promotion in Mexico would provide important health benefits. At the national level, Mexico could consider using sustainable transport policies as a tool to promote public health. Specifically, the support of active transportation through bicycling interventions could promote physical activity, reduce mortality and increase health economic benefits. The attraction of bike users could be supported by bike investments and interventions (e.g., bike lanes, bike parking, and bike-sharing systems), combined with interventions to reduce car use (e.g., parking pricing and reduction, and congestion pricing). To meet ambitious bicycling scenarios in Mexico, strong transport, urban planning, energy, environmental, and health policies should be adopted at national and local levels.

## Figures and Tables

**Table 1:** Input data used in the analysis.

Basal Level of Physical Activity (METs)								
Group 1		Group 2		Group 3				
%	METs	%	METs	%	METs			
19.4	8.95	28.8	26.85	51.8	35.8			
**Urban population** (20 to 64 years old, in 2010)	**Mortality rate** (per 100,000 people, 2017)	**Air pollution** (PM2.5 annual concentration μg/m3)	**Car Speed** (km/h)	**Bike Speed** (km/h)	**Average trips per person per day** (trips/day)		**Average trip length by mode of transport** (km/trip)	
51,718,756	369.36	23.38	30	11.6	3.75		2	
**Traffic fatalities by car per year**	**Traffic fatalities** **per billion kilometers traveled by car**			**Traffic fatalities by bike per year**	**Traffic fatalities** **per billion kilometers traveled by bike**			
	Mean	Lower uncertainty interval	Upper uncertainty interval		Mean	Lower uncertainty interval		Upper uncertainty interval
4704	19.77	9.15	63.09	301	41.21	22.6		133.29

MET: Metabolic Equivalent of Task; PM2.5: Particulate Matter with a dimeter <2.5 μm.

**Table 2:** Results of current and hypothetical bicycling scenarios in Mexico.

Variable	Current Situation	Double Bike Share	Achieving Brazil Levels	Achieving Danish Levels	Achieving Dutch Levels
Bike modal share (%)	1.07	2.13	3	16	27
Total bike trips Mexico (trips/day)	2,068,750	4,137,501	5,818,515	31,032,081	52,366,637
Expected bike trips coming from cars in Mexico (trips/day)	165,500	331,000	465,481	2,482,567	4,189,331
Annual prevented deaths (deaths/year)	9	17	24	129	217
Low uncertanty interval	6	11	16	84	142
Upper uncertanty interval	25	49	69	370	625
Annual economic benefit on mortality (US $/year)	1,897,920	3,584,960	5,061,120	27,203,520	45,760,960

**Table 3:** Policy recommendations to support bicycling in Mexico.

Bicycling
Rapidly implement bicycling infrastructure on a large scale
Implement bike share programs in medium and large-size cities, prioritizing connections to public transport
Prioritize bicycling infrastructure design based on safety, accessibility, connectivity, and aesthetics
Implement and enforce laws and regulations to prioritize bicycling safety
Support bicycling through fiscal and economic incentives and information campaigns
Support open streets in large and medium-sized cities
**Motorize transport**
Eliminate policies that subsidize additional motor vehicle use, such as minimum parking requirements, free on-street parking, and fuel subsidies
Implement motorize transport policies that consider their negative externalities, such as congestion pricing or vehicle kilometers traveled fees
Invest fuel taxes, driving fees, and other transport-system revenues in sustainable transport.
Reduce speed limits to support traffic safety
**Urban planning**
Coordinate metropolitan transport and land-use plans, aiming that all new investments result in more bicycling and fewer trips by motorized vehicles
Support transit-oriented and mixed-use developments Implement car-free streets and neighborhoods
Support a network of green and blue spaces that help to connect bicycling infrastructure
Prioritize a universal design aiming for more inclusive and equitable use of public space and transport networks
**Environment**
Develop laws and regulations that protect the population from harmful air pollution and traffic noise levels
Increase awareness of air pollution sources, especially from motorized vehicles in urban settings
Implement stricter air pollution and noise emission limits from motorized vehicles
Reduce bicyclist air pollution exposure by prioritizing bike infrastructure away from emission sources, such as motorized vehicles
**Public health**
Consider bicycling as a health promotion and prevention tool
Support active transport policies to improve traffic safety
Support active transport policies to improve air quality and reduce noise pollution
Increase collaboration with urban and transport planners
Design public health campaigns to support a healthy lifestyle through active transportation

## Data Availability

Publicly available datasets were analyzed in this study. This data can be found in the references, supplemental material and here: [https://itdpdotorg.wpengine.com/wp-content/uploads/2015/11/A-Global-High-Shift-Cycling-Scenario_Nov-2015.pdf accessed on 1 March 2021] [https://www.who.int/airpollution/data/cities/en/ accessed on 1 March 2021] [https://population.un.org/wup/Publications/Files/WUP2018-Report.pdf accessed on 1 March 2021] [https://www.itf-oecd.org/sites/default/files/docs/irtad-road-safety-annual-report-2019.pdf accessed on 1 March 2021]

## Supplementary Materials

The following are available online at https://www.mdpi.com/1660-4601/18/5/2300/s1, Figure S1. Physical activity model; Figure S2. Dose response functions (DRF) for physical activity and all cause mortality; Figure S3 Air pollution model; Figure S4. Traffic fatality model; Table S1. Relative risk formulas for each model; Table S2. General formulas; Table S3. Air pollution variables; Table S4. Results in annual premature deaths in each scenario by risk factor; Table S5. Sensitivity results in premature deaths prevented each year in each scenario, assuming a 5km bike trip length; Table S6. Sensitivity results in premature deaths prevented each year in each scenario, using the HEAT for walking and cycling V.3* (5 km trip distance).
