# Supplementary material for: Health Impacts of Urban Bicycling in Mexico
Source: Int J Environ Res Public Health. 2021 Feb 26;18(5):2300. doi: 10.3390/ijerph18052300 (PMC7967710; doi:10.3390/ijerph18052300)
Supplement: Supplementary file 1 [file ijerph-18-02300-s001.pdf]

<u>Supplemental Material</u>

Health impacts of Urban Bicycling in Mexico

Rojas-Rueda D PhD [a,]

[a] Department of Environmental and Radiological Health Sciences, Colorado State University, Fort Collins, USA

Address:

David Rojas-Rueda, Colorado State University, 1601 Campus Delivery, 80523 Fort Collins, USA.

Corresponding author: David Rojas-Rueda, Colorado State University, Environmental Health Building, 1601 Campus Delivery, 80523 Fort Collins, USA, Phone: (970) 491-7038 Fax: (970) 491-2940; E-mail address: David.Rojas@colostate.edu (David Rojas-Rueda)

Keywords: Biking; transport; Mexico; health impact assessment; environmental health.

Table of Contents

Section 1: Methods 3
Figure S1. Air pollution model 3
Figure S2. Traffic fatality model 4
Figure S3. Physical activity model 5
Table S1. Relative risk formulas for each model. 6
Table S2. General formulas. 7
Table S3. Air pollution variables 8
Figure S4. Dose response functions (DRF) for physical activity and all cause mortality. 9
Section 2: Results 10
Table S4. Results in annual premature deaths in each scenario by risk factor. 10
REFERENCES 11

Section 1: Methods

Figure S1. Air pollution model

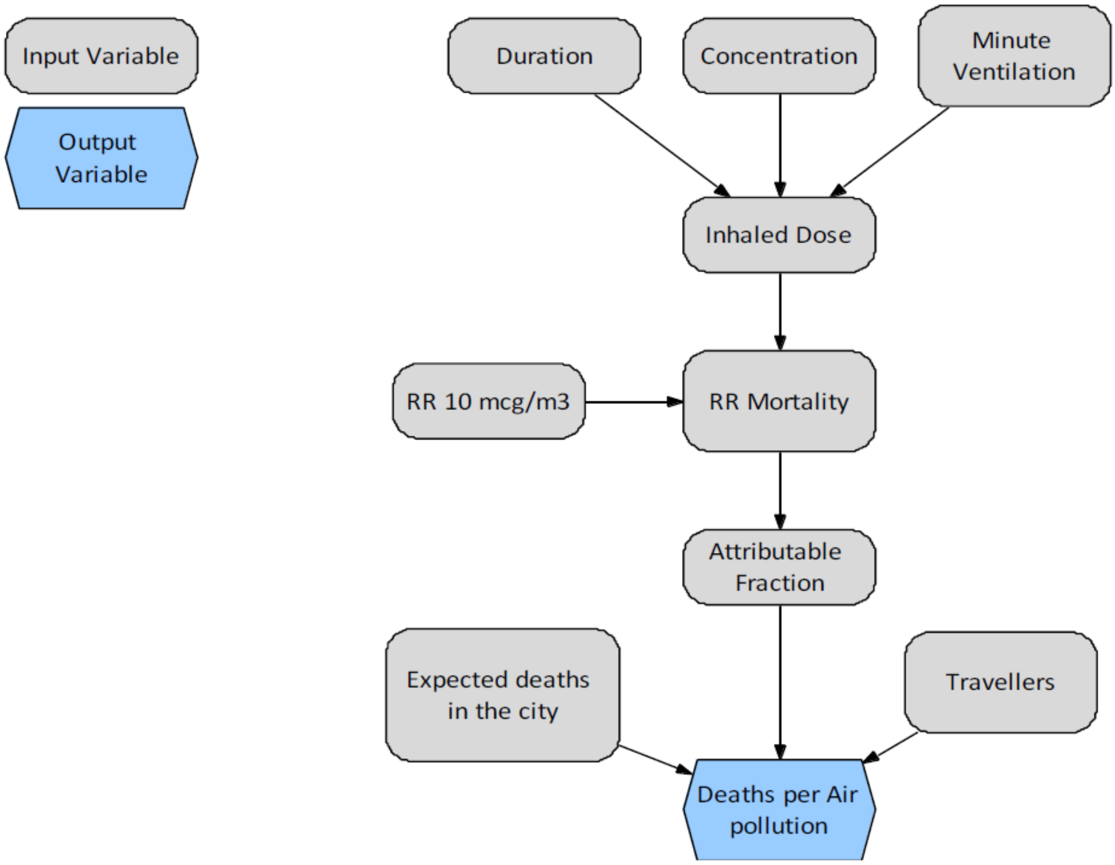

RR: Relative Risk of all-cause mortality.
RR10: average adjusted relative risk of all-caused mortality for a 10µg/m3 change of pollutant.

Figure S2. Traffic fatality model

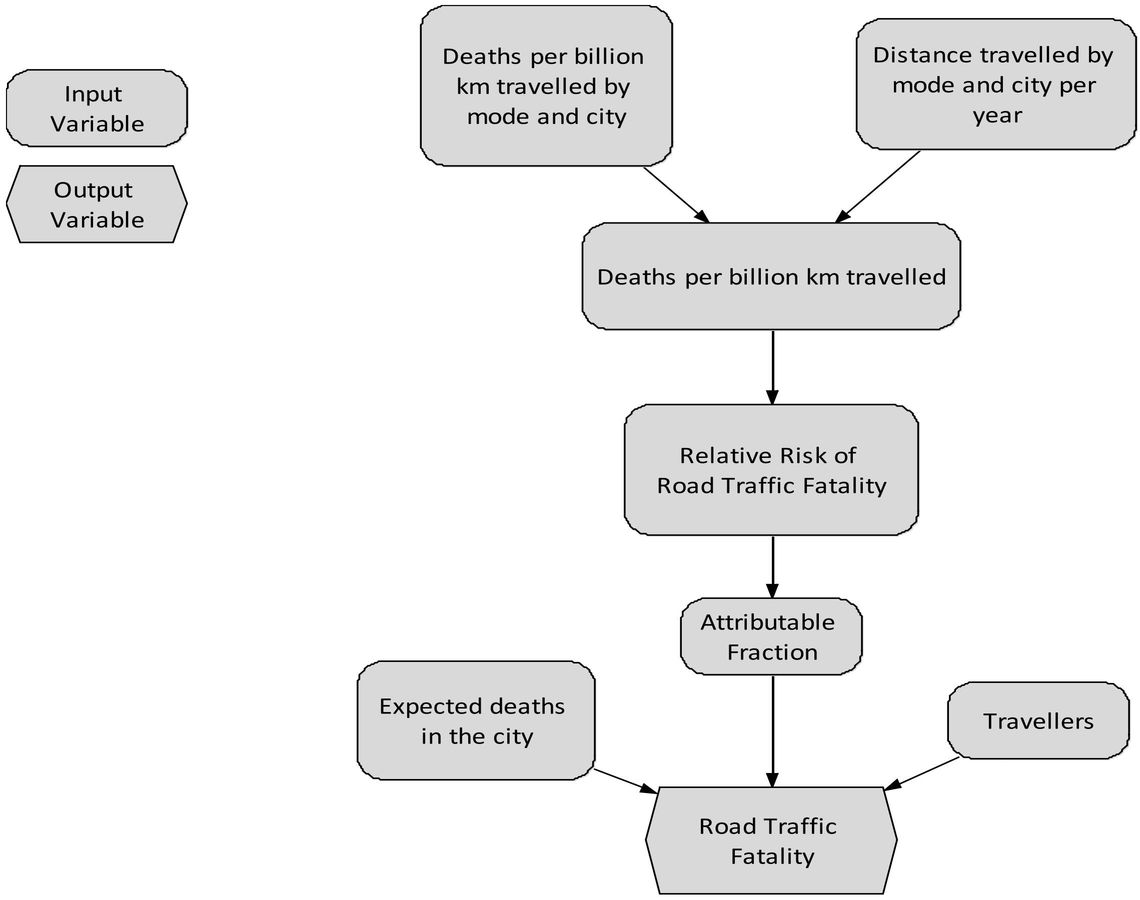

Figure S3. Physical activity model

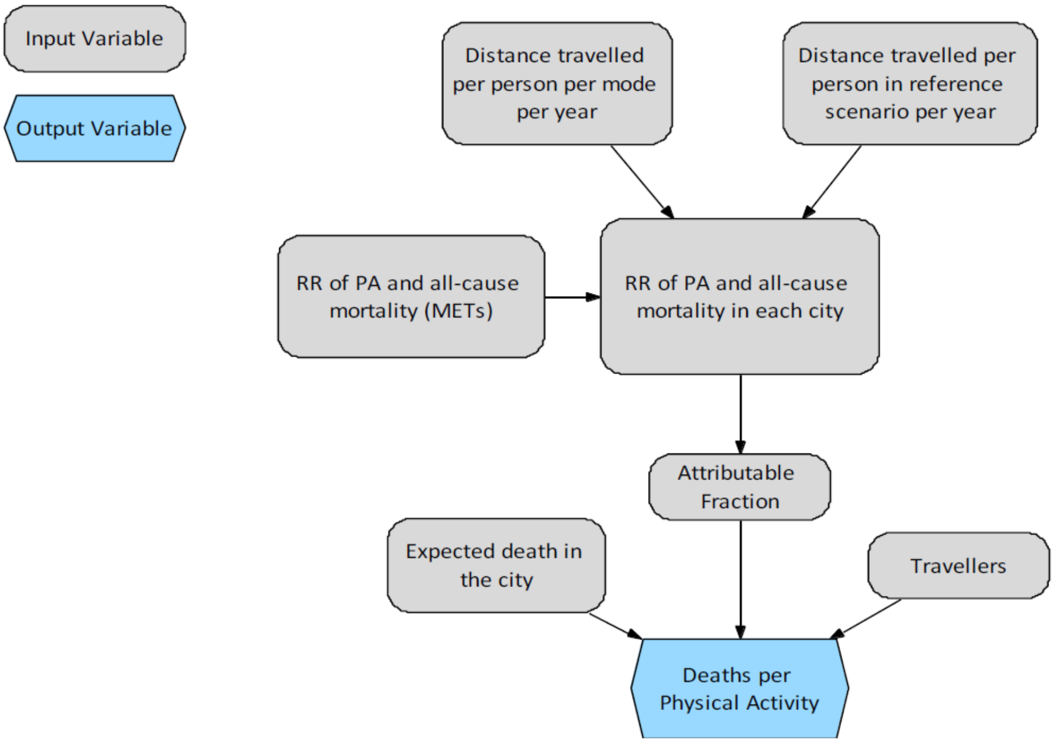

RR: Relative Risk of all-cause mortality.

Table S1. Relative risk formulas for each model.

Relative Risk (RR)	
Physical Activity	RR^(METs^0.25) [a]
Traffic accidents	$\frac{\text{Deaths in the population + (Deaths in Bike - Deaths in car)}}{\text{Deaths in population}}$ [b]
Air Pollution	Exp[Ln (RR_{10}) * $\left(\frac{\text{Equivalent change}}{10}\right)$] [c]

[a] 0.81 per 8.6 METs.

[b] Used deaths per year; deaths in bike and car according with deaths per billion km travelled and distance travelled in each mode.

[c] This RR was calculated for each pollutant, with equivalent change and RR_{10} specific for PM2.5; RR_{10}= average adjusted relative risk of all-caused mortality for a 10μg/m^3 change of pollutant.

Table S2. General formulas.

**Attributable Fraction among exposed**	$AF_{exp} = \frac{(RR-1)}{RR}$
**Mortality rate in Mexican Biking population**	Mortality rate in Mexico * Biking population
**Mortality due to exposure**	Mortality rate in Mexican Biking population * AF_{exp}
**Deaths per billion kilometers traveled**[a]	(Number of fatalities [b] * Kilometers traveled per year) * 1 billon
**Inhaled dose (µg/day)** [c]	Minute ventilation(m^3/h) * Duration(h/day) * Concentration(µg/m^3)
**Total dose (µg/day)** [c]	Inhaled dose during Sleep + Rest + Transport
**Equivalent change (µg/m^3)** [c]	$\left(\left(\frac{\text{Total dose in bike}}{\text{Total dose in car}}\right) -1\right)$ * Mean concentration of pollutant

[a] This formula was calculated for each mode of transport.

[b] The number of fatalities used was the annual average of fatalities per mode in Mexico.

[c] The input data in this formula was weighted by the 307 days a year and calculated for PM2.5.

Table S3. Air pollution variables

	Concentration (μg/m³)	Minute ventilation (m³/hr) [a]	Activity duration (hrs)	Inhaled Dose during each activity on a day (μg)		Total inhaled dose in one day (μg)[c]	
	PM2.5*			PM2.5*		PM2.5*	
Sleep	23.38	0.32	8	59			
Rest	23.38	0.36	15 [b]	126			
Car	46.76	0.72	0.21	7		192	
Bike	38.34	2.47	0.35	33		218	

*PM2.5: Particulate matter les than 2.5 micrometer.

[a] Minute ventilation in bike is calculated using a random population distribution and algorithms developed by the EPA (de Nazelle et al. 2009) from average METs measured for [Bike, car, rest] = [6, 2, 1]. Uncertainty based on data.

[b] Number of hours remaining to reach 24 hours in a day (ie. to the 15hr add 0.79hr for the car scenario and 0.65hr for the bike scenario).

[c] Total inhaled dose is calculated assuming activity durations and minute ventilation for the car scenario and the bike scenario, weighed for 307 days that are considered to be travelling.

Figure S4. Dose response functions (DRF) for physical activity and all cause mortality.

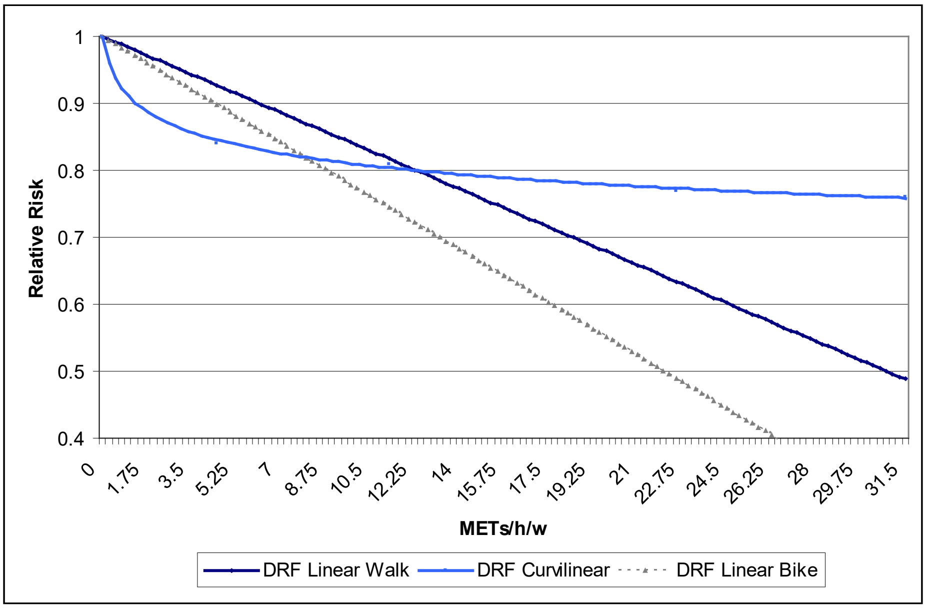

* METs/h/w: Metabolic Equivalent of Task per hour per week; DRF: Dose Response Function; Curvilinear DRF from a meta-analysis for physical activity and all-cause mortality (Woodcock J. 2010); Linear Walk DRF from a meta-analysis reported in HEAT for walking (WHO, 2010); Linear Cycling DRF from HEAT for cycling (Andersen L, 2000).

Section 2: Results

Table S4. Results in annual premature deaths in each scenario by risk factor.

	Current situation	Double bike share	Achieving Brazil levels	Achieving Danish levels	Achieving Dutch levels
**Traffic fatalities**	2	4	6	32	53
**Air Pollution**	1	2	3	19	31
**Physical Activity**	-12	-24	-34	-179	-302
**Total**	-9	-17	-24	-129	-217

Table S5. Senstvityty results in premature deaths prevented each year in each scenario, assuming 5km bike trip length.

	Current situation	Double bike share	Achieving Brazil levels	Achieving Danish levels	Achieving Dutch levels
**Total**	15	30	43	228	384

Table S6. Senstvityty results in premature deaths prevented each year in each scenario, using the HEAT for walking and cycling V.3* (5km trip distance).

	Current situation	Double bike share	Achieving Brazil levels	Achieving Danish levels	Achieving Dutch levels
**Total**	21	41	58	309	522

* http://old.heatwalkingcycling.org/index.php

REFERENCES

Andersen,L.B., Schnohr,P., Schroll,M., and Hein,H.O. (2000) All-cause mortality associated with physical activity during leisure time, work, sports, and cycling to work. *Arch. Intern. Med.* **160:** 1621-1628.

de Nazelle A, Rodriguez DA, Crawford-Brown D. The built environment and health: impacts of pedestrian-friendly designs on air pollution exposure. *Sci.Total Environ.* 2009;**407**:2525-35.

WHO Europe . HEAT for Walking, Meeting Report. 5-9-2010. Oxford UK, World Health Organization Europe.

Woodcock,J., Franco,O.H., Orsini,N., and Roberts,I. (2010) Non-vigorous physical activity and all-cause mortality: systematic review and meta-analysis of cohort studies. *Int. J. Epidemiol.*
